# Incidence and Survival Analysis of Gastrointestinal Stromal Tumors in Shanghai: A Population-Based Study from 2001 to 2010

**DOI:** 10.1155/2014/834136

**Published:** 2014-04-23

**Authors:** Minzhi Lv, Chunxiao Wu, Ying Zheng, Naiqing Zhao

**Affiliations:** ^1^Department of Biostatistics, Fudan University, Shanghai 200032, China; ^2^Shanghai Municipal Center for Disease Control and Prevention, Shanghai 200336, China

## Abstract

*Objectives.* A population-based study was undertaken to investigate the epidemiological features of gastrointestinal stromal tumors (GISTs) in Shanghai, especially the incidence and the preliminary exploration of survival. * Methods.* A total of 1923 patients with GISTs diagnosed from 2001 to 2010 in Shanghai were reviewed. The annual incidence and overall survival of GISTs were calculated; Cox proportional hazards' regression was used to analyze several prognostic factors. * Results.* The average crude incidence of GISTs was 2.11 per 100,000 between 2004 and 2008, and the age-standardized incidence was 1.28 per 100,000. The incidence increased gradually from 2004 to 2008. In addition, 57% of cases had GIST in the stomach and 33% in the intestine. The 5-year overall survival of GISTs was 86.98%. The Cox regression analysis showed older age (≥65 yr versus <40 yr, HR = 5.085; (40, 65) yr versus <40 yr, HR = 1.975), male gender (HR = 1.474), and tumor locations (intestinal versus stomach, HR = 1.609) were predictors of its mortality. * Conclusion.* GISTs, mainly occurring in the stomach, are more common in elderly population, with an increasing incidence from 2004 to 2008. Older age, male gender, and tumor locations are risk factors for its mortality.

## 1. Introduction

Gastrointestinal stromal tumor (GIST), a kind of rare tumor, is the most common mesenchymal tumor in the gastrointestinal tract [1–4]. The concept of stromal tumor was first proposed by Mazur and Clark in 1983. Most GISTs have been previously classified as smooth-muscles tumors (leiomyoma, leiomyosarcoma) or schwannoma due to the incomplete understanding of its origin and differentiation [1, 5]. Presently, it is considered that GISTs originate from stem cells that differentiate toward interstitial Cajal expressing tyrosine kinase receptors KIT or platelet-derived growth factor receptor alpha (PDGFRA) [1, 2, 6]. GISTs are significantly immunopositive for CD117, which is crucial for the diagnosis of GIST, and the positive rate of CD117 may be up to 95% [1–3].

The frequent symptoms of GIST patients include gastrointestinal bleeding, abdominal pain, and abnormal masses, while some with small GISTs are asymptomatic [5, 7]. Currently, on the basis of several population-based studies, the annual incidence of GIST ranges between 1 and 2 per 100,000 [8–14]. Complete surgical resection has been the treatment of choice for localized resectable GISTs [15]. With the discovery of tyrosine kinase inhibitor, imatinib mesylate (Glivec/Gleevec, Novartis), the treatments for and outcome of patients with GISTs have changed dramatically. Since 2000, the safety and efficacy of imatinib in the treatment of metastatic GISTs have been confirmed by many clinical trials [16–18], and a phase III clinical trial has also proved that the adjuvant imatinib is safe and can significantly improve the recurrence-free survival when compared with placebo after the resection of localized, primary GISTs [19]. Currently, imatinib has become a standard first-line drug for the treatment of GISTs and is mainly recommended for patients with unresectable, recurrent, or metastatic GISTs [1, 3, 15]. Additionally, the emergence of another tyrosine kinase inhibitor named sunitinib malate is also of great significance. At present, it has been approved by the U.S. FDA to be used in the second-line therapy.

Although numerous population-based studies in western countries have been published on the epidemiology and prognosis of GISTs, there are few studies undertaken to investigate the characteristics of GISTs in Asian countries. In Mainland China, since the cancer registry and management system have not been implemented nationally, it is difficult to conduct a nationwide study and completely collect data. Therefore, the incidence, survival, and prognostic factors of GISTs in China have not been clear. To figure out these problems, data collected from the cancer registry and management system in Shanghai were used to evaluate the epidemiological features and explore the overall survival of GISTs in China.

## 2. Materials and Methods

### 2.1. Data Sources

Data in this study was mainly collected from the Shanghai cancer registry and management system. This system, established in 2002, is managed by Shanghai Municipal Center for Disease Control and Prevention (SCDC) to identify all new cases. Shanghai is the first area to carry out this population-based cancer registry system. According to the Cancer Registry Regulations, the system aimed to identify malignancies in Shanghai residents. Considering those misclassified cases or benign tumors which are not reported to the SCDC, this study also collected records of GISTs as a supplement from Shanghai Medical Insurance Bureau, and the history records from five hospitals (Zhongshan Hospital, Ruijin Hospital, Changhai Hospital, Shanghai 1st People's Hospital, and Shanghai 6th People's Hospital) were reviewed to confirm these cases. The five hospitals are hospitals where a majority of patients with GISTs are treated. We reviewed the patients' ID, birth date, date of diagnosis, and date of reporting, and then we matched these data comprehensively and removed duplicate data carefully. Finally, a total of 1923 cases were collected, including their medical information at baseline, pathological diagnosis, and survival. The demographics in this study were obtained from the Shanghai Statistical Yearbook of 2011 [20] on the official website of Shanghai Statistics Bureau. The WHO world standard population distribution used was obtained from the WHO official website [21].

### 2.2. Study Population

All cases in the SCDC cancer registry and management system were identified by the coding system of the International Classification of Diseases for Oncology, 3rd Revision (ICD-O), from the World Health Organization. In order to confirm the diagnosis and examine the impact of changes on coding, we also retrospectively collected and checked records of diagnosis and pathological reports of these cases. Finally, a total of 1923 patients who were diagnosed from 2001 to 2010 were recruited to calculate the overall survival (OS) in this study. OS was calculated from the date of diagnosis until death or the end of study (December 31, 2010). As cases collected in 2001–2003 and in 2009-2010 were much fewer than in other years, the incidence was determined in 2004–2008 in the present study. There were 1443 GIST cases diagnosed from 2004 to 2008.

According to the tumor locations, all cases were categorized into three groups: GISTs of the stomach, GISTs of the intestine, and GISTs of other organs. The intestines mentioned above include small intestine (duodenum, jejunum, and ileum), colon, and rectum. The other organs mentioned above include esophagus, peritoneum, and organs adjacent to the gastrointestinal tract.

### 2.3. Statistical Methods

Incidence was calculated as crude incidence (per 100,000) from 2004 to 2008, Moreover, the crude incidence specific to gender and tumor location was calculated. We also calculate the age-standardized incidence, according to the WHO world standard population distribution [21]. Because of low incidence of GISTs, the GISTs were regarded as having an approximate Poisson distribution. Therefore, we fitted a Poisson regression including gender, year of diagnosis, and tumor location as variables.

In this study, 1923 cases collected between 2001 and 2010 were stratified by gender, age, and tumor location to calculate the 5-year OS. OS was calculated using the Kaplan-Meier method. The log-rank test was used to compare various groups. Cox proportional hazards model was used for univariate and multivariate analysis to assess the prognostic significance of these variables. Cox proportional hazards regression analysis was employed to estimate the hazard ratios and 95% confidence intervals (CI) of prognosis factors.

All calculations were performed with the STATA version 11.0. A value of *P* < 0.05 was considered statistically significant.

## 3. Results

In the present study, 1923 cases of GIST were diagnosed between 2001 and 2010. The mean age at diagnosis was 60.05 ± 12.98 years (range: 12–87 years). The number of cases fluctuated significantly among different age groups. Over 80% of cases (84.34%) were diagnosed in individuals at an age of older than 40 years and less than 1% (0.68%) at an age of younger than 25 years (*n* = 1732). Among GISTs patients, 49.60% were men and 50.40% were women (*n* = 1742).

### 3.1. Incidence of GISTs between 2004 and 2008

**Figure 1:**
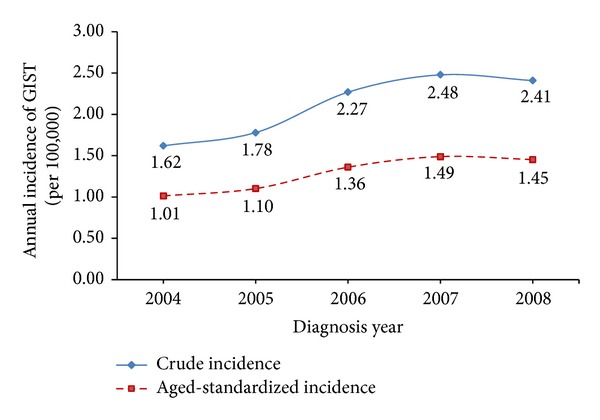
Plots for the annual crude incidence and world age-standardized incidence of GISTs between 2004 and 2008 (per 100,000).

There were 1,443 GISTs patients diagnosed between 2004 and 2008. The average incidence of GIST was 2.11 per 100,000 between 2004 and 2008, and the average age-standardized incidence was 1.28 per 100,000 (Table 1). Our results showed that the incidence of GIST increased steadily from 1.62 per 100,000 in 2004 to 2.48 per 100,000 in 2007. In 2008, the annual incidence decreased slightly to 2.41 per 100,000. Moreover, the world age-standardized incidence had the same trend (Figure 1).

Among these cases, the male-female ratio was 1 : 1.04 (*n* = 1280). The average incidence of GISTs was 1.91 per 100,000 for women, which was a little higher than in men (1.83 per 100,000). The annual incidence in men rose from 1.40 per 100,000 in 2004 to 2.22 per 100,000 in 2007 and declined slightly to 2.05 per 100,000 in 2008. From 2004 to 2008, the annual incidence in women increased over year (Table 1).

In the present study, 57.3% of GISTs were found in the stomach (*n* = 827), 32.9% in the intestine (*n* = 475), and 9.8% in other organs (*n* = 141). The average incidence of GISTs in the stomach and in the intestine was 1.22 per 100,000 and 0.695 per 100,000; respectively, and the incidence of GISTs in the stomach was the highest.

We fitted a Poisson regression including gender, year of diagnosis, and tumor locations as variables. Results demonstrated that the incidence of GISTs increased over year (*P* = 0.000) (see Table 2). The incidence of GISTs was comparable between males and females (*P* = 0.452). Significant difference was noted in the annual incidence of GISTs among patients with different tumor locations (*P* = 0.000). The quantity exp⁡⁡(*β*_3_) = 0.5409 gave the hazard ratio when comparing the incidence of GISTs in the intestine with that in the stomach, and the hazard ratio of other organs and the stomach was exp⁡⁡(*β*_4_) = 0.1193, which implied the stomach was the most common primary organ for GISTs.

### 3.2. Survival of GIST Patients between 2001 and 2010

In our study, a total of 1923 GIST patients were collected from the date of diagnosis until death or December 31, 2010. The 1-year, 3-year, and 5-year OS were 94.59%, 89.79%, and 86.98% (95% CI: 85.2–88.6), respectively. The 5-year OS for men (*n* = 864) and women (*n* = 878) was 83.37% and 87.68%, respectively. The 5-year OS for patients older than 65 years (*n* = 686) was 78.49%, which was much lower than that in patients younger than 65 years. The 5-year OS was 89.31% for patients with GISTs in the stomach (*n* = 1126) and 84.24% for those with GISTs in the intestine (*n* = 616). The log-rank test results showed that each variable was significantly correlated with the survival (Table 3).

The Cox multivariate regression analysis is shown in Table 4. Male gender (HR = 1.47, *P* = 0.004), older age (age ≥ 65, HR = 5.09,

*P* = 0.000), and tumor location (intestine, HR = 1.61, *P* = 0.001; other organs, HR = 1.99, *P* = 0.001) were significant unfavorable prognostic factors in GISTs patients.

## 4. Discussion

### 4.1. Incidence

This is the first population-based study to describe the epidemiological characteristics of GISTs in Mainland China. Our results showed the crude incidence of GISTs between 2004 and 2008 was 2.11 per 100,000 and the age-standardized incidence was 1.28 per 100,000. Compared with previous studies in Europe and North America, the crude incidence in China (2.11 per 100,000) was higher than that in Canada (0.51–0.96 per 100,000), France (0.85–1.0 per 100,000), Italy (1.42 per 100,000), and Sweden (1.45 per 100,000) [8, 11, 14, 22]. Moreover, the world age-standardized incidence in our study (1.28 per 100,000) was also higher than that in Iceland (1.1 per 100,000), Italy (0.66 per 100,000), and Spain (0.65 per 100,000) [9, 11, 12]. However, the crude incidence was close to that reported by another Asian study in Korea (1.6–2.2 per 100,000) [23]. And the age-standardized incidence of another population-based study in Taiwan (1.13–1.97 per 100,000) was similar to our results [24].

The higher incidence in our study might be explained by an improved understanding of the pathobiology of GISTs. For many years, GISTs were frequently misdiagnosed until the characteristic receptor tyrosine kinase type III (CD117/kit protein) for GIST was discovered in 1998 [25]. However, a large proportion of patients in previous studies were diagnosed before 2000. During that period, the diagnostic criteria and the use of CD117 immunohistochemistry were not proposed. Given these reasons, it is reasonable that the incidence we estimated is higher than many previous studies.

In addition, the higher incidence in the present study might be also related to races, which means that Asians are more susceptible to GISTs. The results of Tran et al. indicated that the incidence among different race groups differed significantly, and the incidence in Whites was lower than in other races [13]. Moreover, differences in lifestyle, especially the diet, and environmental factors may also account for the difference in incidence. Further studies are required to confirm these risk factors.

Our study showed that the crude incidence increased gradually by year (*P* < 0.0001), from 1.62 to 2.48 per 100,000. Although the true incidence of GISTs increases over year, this may be related to the progress in the pathology of GISTs, which leads to proposal of reliable diagnostic criteria and wide use of CD117 immunohistochemical staining. Actually, the similar result has also been described in other studies [10, 22–24, 26]. Goettsch et al. [10] concluded that the incidence of GISTs increased from 2.1 per million in 1995 to 12.7 per million in 2003, which was related with the availability of specific diagnostic marker CD-117 antigen. Thus, it is reasonable to conclude the increasing trend of incidence.

However, the true incidence should be higher than what we calculated. Some patients with small or clinically insignificant GISTs are always asymptomatic, which frequently are detected accidentally at autopsy or during surgery for other reasons. The study conducted by Nilsson et al. showed that about 70% of GISTs were diagnosed depending on symptoms, 20% of GISTs were accidentally found at surgery, and the remaining were found at autopsy [8]. Another Canada population-based study also drew the similar conclusion [22]. Moreover, a German study reported small (<10 mm) GISTs in 22.5% of the elders (>50 years) were accidentally detected on autopsies [27]. Therefore, the accurate incidence of asymptomatic GISTs could not be evaluated at present and further studies are warranted to develop more diagnostic techniques.

In this study, our results showed that the male to female ratio was 1 : 1.04, and the incidence of GISTs in females was a little higher than that in males (1.91 per 100,000 versus 1.83 per 100,000); but there was no statistical significance (*P* = 0.4520). Although this result is similar to the study in Sweden [8], it is still controversial. In the study of Iceland [9], results showed a male predominance of GISTs, which was consistent with the studies from United States [13], Korea [23], and Taiwan [24]. It is noteworthy that in the United States study, the incidence of malignant GISTs was significantly higher in males.

### 4.2. Survival

In this study, the 5-year OS of GISTs was 86.98%. When compared with other studies in years before the introduction of imatinib, the 5-year OS in this study was significantly increased [11, 12, 28], which can be attributed to the wide use of tyrosine kinase inhibitor, imatinib. On the other hand, this can also be caused by the heterogeneity of study populations. For example, some studies only included malignant GISTs, but others might also include benign GISTs.

The impacts of gender and tumor location on survival are still controversial. Some studies argued that they were not prognostic factors of GISTs [8, 11, 29]. However, in our study, the Cox multivariate analysis revealed that the age at diagnosis, gender, and tumor location were significant independent predictors of survival of GIST patients. Male gender was identified as a negative predictor of survival (HR = 1.47), which was consistent with findings in other studies [9, 23, 24, 30]. Moreover, results also revealed that patients with stomach GISTs had better survival, which signified that GISTs in the stomach were less aggressive. This was supported by other studies [24, 31]. Actually, the recent update on NCCN guidelines suggested anatomic site as an additional prognostic factor [4].

## 5. Limitations and Conclusion

There are limitations in this study. First, with the limited information and the inadequacy of study design, we could not collect more information such as the tumor size, treatments, symptoms, or environmental factors. We expect to collect more information in future studies or conduct a cohort study to explore these risk factors and prognostic factors. Second, in the present study, we only calculated the incidence of GISTs in recent five years, which showed an increasing trend. This trend is mainly explained by the improved understanding of the pathology of GISTs, which may be biased against the tendency of true incidence. Thus, it is necessary to investigate the incidence of GISTs in a long time.

In conclusion, GISTs have a low incidence; the elderly are more likely to develop GISTs, and the stomach is the most common site of GISTs. There is an increasing trend in the incidence of GISTs from 2004 to 2008, which is mainly attributed to the improved understanding of GIST pathology and the wide use of CD117 immunohistochemical staining. Older age, male gender, and tumor location (intestinal, others) are risk factors of mortality in GIST patients.

## Figures and Tables

**Table 1:** Annual crude incidence of GISTs between 2004 and 2008 (per 100,000).

Year of diagnosis	Incidence	Gender	
	(per 100,000)	Male	Female
2004	1.62	1.40	1.43
2005	1.78	1.61	1.50
2006	2.27	1.88	2.18
2007	2.48	2.22	2.21
2008	2.41	2.05	2.24

Overall	2.11	1.83	1.91

**Table 2:** Poisson regression for multivariate analysis of GIST incidence.

	Coefficient $\hat{\beta }$	IRR	*P* value	95% CI	
Gender					
Female	Reference				
Male	−0.0421	0.9588	0.4520	0.8593	1.0698
Diagnosis year	0.1130	1.1196	0.0000	1.0767	1.1643
Location					
Stomach	Reference				
Intestine	−0.6146	0.5409	0.0000	0.4801	0.6093
Others	−2.1259	0.1193	0.0000	0.0961	0.1481

**Table 3:** 5-year overall survival and 95% CI for GISTs between 2001 and 2010, calculated with Kaplan-Meier method, and the log-rank test.

	*N*	5-year survival	*P* value	95% CI	
Overall	1923	0.8698		0.8517	0.8859
Gender					
Male	864	0.8337	0.0056	0.8038	0.8594
Female	878	0.8768		0.8487	0.8999
Age					
<40	110	0.9496	<0.0001	0.8826	0.9788
[40, 65)	936	0.9048		0.8806	0.9244
≥65	686	0.7849		0.7475	0.8175
Location					
Stomach	1126	0.8931	0.0063	0.8702	0.9121
Intestine	616	0.8424		0.8074	0.8716
Others	181	0.8220		0.7508	0.8745

**Table 4:** Cox proportional hazards model with multivariate analysis.

	Hazard ratio	*P* value	Standard Error	95% CI	
Age					
<40	Reference				
[40, 65)	1.9754	0.1070	0.8354	0.8623	4.5250
**≥**65	5.0853	0.0000	2.1284	2.2390	11.5499
Gender					
Female	Reference				
Male	1.4740	0.0040	0.1996	1.1303	1.9221
Location					
Stomach	Reference				
Intestine	1.6092	0.0010	0.2322	1.2127	2.1353
Others	1.9870	0.0010	0.4120	1.3235	2.9832
